# Self-Reported Diabetes Mellitus and Tooth Extraction Due to Periodontal Disease and Dental Caries in the Japanese Population

**DOI:** 10.3390/ijerph18179024

**Published:** 2021-08-27

**Authors:** Seitaro Suzuki, Naoki Sugihara, Hideyuki Kamijo, Manabu Morita, Takayuki Kawato, Midori Tsuneishi, Keita Kobayashi, Yoshihiro Hasuike, Tamotsu Sato

**Affiliations:** 1 Department of Epidemiology and Public Health, Tokyo Dental College, Kanda-Misaki-cho, Chiyoda-ku, Tokyo 101-0061, Japan; sugihara@tdc.ac.jp; 2 Department of Social Security for Dentistry, Tokyo Dental College, Kanda-Misaki-cho, Chiyoda-ku, Tokyo 101-0061, Japan; kamijohideyuki@tdc.ac.jp; 3 8020 Promotion Foundation, 4-1-20 Kudankita, Chiyoda-ku, Tokyo 102-0073, Japan; tsuneishi_mi@jda.or.jp (M.T.); ktadent@sa2.so-net.ne.jp (K.K.); hasuike@khaki.plala.or.jp (Y.H.); tamosato-dent@k-2inc.jp (T.S.); 4 Department of Preventive Dentistry, Okayama University Graduate School of Medicine, Dentistry and Pharmaceutical Sciences, Shikata-cho, Kita-ku, Okayama 700-8530, Japan; mmorita@md.okayama-u.ac.jp; 5 Department of Oral Health Sciences, Nihon University School of Dentistry, 1-8-13 Kanda-Surugadai, Chiyoda-ku, Tokyo 101-8310, Japan; kawato.takayuki@nihon-u.ac.jp; 6 Japan Dental Association Research Institute, 4-1-20 Kudankita, Chiyoda-ku, Tokyo 102-0073, Japan; 7 Japan Dental Association, 4-1-20 Kudankita, Chiyoda-ku, Tokyo 102-0073, Japan

**Keywords:** dental caries, diabetes mellitus, periodontal disease, disease interactions

## Abstract

Diabetes mellitus is closely related to oral health. We aimed to determine the relationship between diabetes mellitus and tooth extraction due to periodontal disease and dental caries. Japan’s second nationwide survey data collected from 4 June to 10 June 2018 was used to identify reasons for tooth extraction among patients aged > 40 years. General dentists collected information on patients who underwent tooth extraction procedures, and the presence of diabetes mellitus was determined through interviews. Multivariable logistic regression was performed to investigate the relationship between diabetes mellitus and the reasons for tooth extraction, including periodontal disease and dental caries. In total, 2345 dentists responded to the survey (response rate 44.8%). We analyzed data on 4625 extracted teeth from 3750 patients (1815 males and 1935 females). Among patients with self-reported diabetes mellitus, 55.4% had extractions due to periodontal disease compared to 46.7% of such extractions among those without self-reported diabetes mellitus. Self-reported diabetes mellitus was significantly associated with tooth extraction due to periodontal disease. No significant differences were observed in dental caries according to self-reported diabetes mellitus status. This study provides further evidence of a significant association between diabetes mellitus and tooth extraction due to periodontal disease.

## 1. Introduction

Diabetes mellitus is closely associated with oral health [1,2]. High glucose levels, advanced glycation end-products, and reactive oxygen species in the periodontal tissues of patients with diabetes mellitus cause a host response that leads to inflammation associated with the development of periodontal disease [3]. Additionally, Borgnakke et al. showed that periodontal disease might adversely affect diabetes mellitus by systematically reviewing 17 studies [4]. They found that people with periodontal disease have a greater risk of poor blood glucose levels and diabetes-related complications among people with diabetes mellitus. Therefore, a bidirectional association was described between diabetes mellitus and periodontal disease [5].

Several studies have also reported an association between dental caries and diabetes mellitus [6,7,8]. De Lima et al. [7] reported that dental caries was more prevalent among patients with diabetes mellitus than among healthy controls. Lattie et al. [8] found that the Decayed, Missing, and Filled Teeth index in patients with diabetes mellitus was twice as high as that in patients without diabetes mellitus.

The endpoint of periodontal disease and dental caries is tooth extraction. Considering that periodontal disease and dental caries are more common in patients with diabetes mellitus, it is reasonable to assume that patients with diabetes mellitus experience more tooth loss than those patients without diabetes mellitus. Several studies have reported tooth loss in patients with diabetes mellitus [9,10,11]. However, few studies have reported the reasons for tooth extraction in patients with diabetes mellitus. Revealing the relationship between diabetes mellitus and tooth extraction due to periodontal disease and dental caries would provide further evidence of a relationship between diabetes mellites and oral health. We hypothesized that diabetes mellitus is associated with tooth extractions due to periodontal disease and dental caries.

## 2. Materials and Methods

### 2.1. Study Design and Data Collection

This was a cross-sectional study. We used tooth extraction data from Japan’s second nationwide survey data collected by the 8020 Promotion Foundation between 4 June and 10 June 2018. The survey was conducted to determine the national situation of tooth loss and its causes. Survey participants were dentists affiliated with the Japan Dental Association. A sampling target of 5000 out of 52,449 affiliated dentists (as of 1 April 2018) was set based on a previous study [12]. One-tenth sampling was performed based on the number of dentists in each prefecture. As a result, 5250 dentists participated in this study. The dentists recorded the reasons for any permanent tooth extractions performed during the observation period. Detailed information on the characteristics of dentists has been reported elsewhere [13].

We analyzed the relationship between diabetes mellitus and the reasons for tooth extraction in patients aged over 40 years because there is a high prevalence of diabetes mellitus among individuals in that age group in Japan [14].

This study was approved by the Ethics Committees of Tokyo Dental College and the Japanese Association for Dental Science (approval numbers 1027 and 018, respectively) and conducted in accordance with the Declaration of Helsinki. The requirement for informed consent was waived because all data were anonymized during the recording of tooth extraction information by the dentists.

### 2.2. Reasons for Tooth Extraction

The reasons for tooth extraction were categorized into six groups, namely dental caries, periodontal disease, fracture, orthodontics, impacted teeth, and others. Dentists determined the main reason for tooth extraction. If teeth were extracted for multiple reasons, the dentists determined the single main reason for tooth extraction. These six groups were then categorized into three groups: dental caries, periodontal disease, and other reasons. To prevent tooth-dependent effects from confounding the results in patients with multiple extractions we excluded cases where some teeth were extracted for one reason and others extracted for a different reason [15]. Extractions of the third molars were also excluded.

### 2.3. Diabetes Mellitus and Other Variables

Laboratory data are ideal for identifying patients with diabetes mellitus. However, this survey was conducted during routine dental care; therefore, laboratory data were not available, and we relied on self-reported diabetes mellitus information. As for the validation of using self-reported diabetes, Goto et al. [16] reported that the sensitivity of self-reported diabetes mellitus was 70.4%.

Dentists collected information regarding the number of teeth before the extraction and the type of extracted tooth. Additionally, smoking status (current and nonsmokers) was assessed through interviews. Health inequalities have been reported in Japan [17]. Therefore, we added each dental clinic’s location as a variable in this study, categorized as urban (20 ordinance-designated cities [18]) or rural (other than ordinance-designated cities).

### 2.4. Statistical Analysis

A chi-squared test or Fisher’s exact test was used to compare the characteristics between the extracted teeth in patients with and without self-reported diabetes mellitus, and to determine the statistical significance of differences in reasons for tooth extraction by oral status. Multivariable logistic regression analysis was performed to investigate the relationship between diabetes mellitus and the reasons for tooth extraction (periodontal disease and dental caries). Four models were tested for each reason for tooth extraction, considering the difference in the relationship by covariates. Model 1 was an unadjusted model applied to evaluate the relationship between diabetes mellitus and each reason for tooth extraction. Model 2 was adjusted for sex, age, and the dental clinic’s location. Model 3 was adjusted for the number of teeth before extraction and the type of teeth, in addition to Model 2. Model 4 was adjusted for smoking status, in addition to Model 3. Multivariable logistic regression analyses were performed using a forced entry method. All statistical analyses were performed using SPSS version 26.0 (IBM Corp., Armonk, NY, USA).

## 3. Results

**Figure 1:**
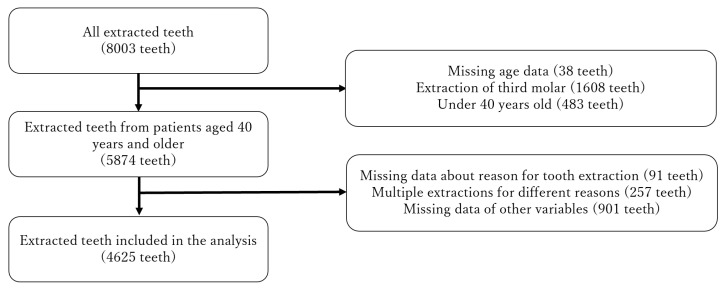
Flow chart summarizing the selection of the extracted teeth for the analysis.

In total, 2345 dentists responded to the survey (response rate, 44.8%). Figure 1 shows the selection of extracted teeth for the analysis. A total of 8003 teeth were extracted from 6541 patients during the observation period; 4625 extracted teeth from 3750 patients (1815 males and 1935 females; mean age, 66.9 ± 11.9 years; age range, 40–97 years) were analyzed in this study. The mean number of extracted teeth per patient was 1.23 ± 0.63 (range, 1–9 extracted teeth per patient).

Table 1 shows the characteristics of the extracted teeth in patients with and without self-reported diabetes mellitus: significant differences between the two groups were observed for sex, age, number of teeth before extraction, type of teeth, and reason for tooth extraction. Male patients comprised 57.6% of patients with self-reported diabetes mellitus, whereas female patients accounted for 52.0% of patients without self-reported diabetes mellitus. An anterior tooth was the most commonly extracted tooth among patients with self-reported diabetes mellitus, whereas a molar was the most commonly extracted tooth among patients without self-reported diabetes mellitus. Periodontal disease was the main cause of tooth extraction in both groups.

Table 2 shows the reasons for tooth extraction by the number of teeth before extraction. Regardless of the number of teeth, periodontal disease was the main reason for tooth extraction. Among patients with at least 20 teeth present, the prevalence of periodontal disease was higher among patients with self-reported diabetes mellitus than among those without diabetes mellitus (48.9% vs. 38.1%). The prevalence of dental caries was relatively similar in the two groups (21.4% vs. 24.1%).

The reasons for tooth extraction are summarized in Table 3. As for the upper anterior teeth, the prevalence of tooth extraction by periodontal disease was higher among patients with self-reported diabetes mellitus than among those without diabetes mellitus (64.8% vs. 49.7%). Meanwhile, as for dental caries, patients without self-reported diabetes mellitus had more tooth extractions due to dental caries than patients with self-reported diabetes mellitus (22.4% vs. 13.0%). Regardless of the type of teeth, periodontal disease was the main reason for tooth extraction.

Associations between self-reported diabetes mellitus and tooth extraction due to dental caries and periodontal disease are shown in Table 4. In the unadjusted model, diabetes mellitus was significantly associated with tooth extraction due to dental caries (odds ratio [OR]: 0.70, 95% confidence interval [CI]: 0.58–0.86). However, after adjusting for sex, age, and location (Model 2), the association was not statistically significant; associations in Models 3 and 4 were also not significant. Meanwhile, diabetes mellitus was significantly associated with tooth loss due to periodontal disease in the unadjusted model (OR: 1.42, 95% CI: 1.17–1.72) as well as the fully adjusted model (OR: 1.23, 95% CI: 1.01–1.50).

## 4. Discussion

In this study, we aimed to identify the relationship between diabetes mellitus and tooth extraction due to periodontal disease and dental caries. The results showed that self-reported diabetes mellitus was significantly associated with tooth extraction due to periodontal disease. However, an association with dental caries was not observed.

A two-way association between diabetes mellitus and periodontal disease has been well established [3,19]. Moreover, uncontrolled diabetes mellitus (glycated hemoglobin [HbA1c] ≥ 6.5%) is associated with severe periodontitis [19]. Greenblatt et al. [20] reported that patients with uncontrolled diabetes mellitus (HbA1c ≥ 7%) experienced more tooth loss than those with controlled diabetes mellitus (HbA1c < 7%). Therefore, an association reported between diabetes mellitus and tooth extraction due to periodontal disease in the current study was expected. Moreover, the strength of association (OR: 1.23) was similar to that in a previous study by Yoo et al. [21], which compared the risk of tooth extraction due to periodontal disease between patients with and without diabetes mellitus (OR: 1.3) using a nationwide database.

Patients with self-reported diabetes mellitus had more tooth extractions due to periodontal disease than those without self-reported diabetes mellitus, among those with ≥20 teeth before extraction (Table 2), and we evaluated whether tooth type was associated with this result. Among the teeth extracted due to periodontal disease, 51.7% were molars in patients with self-reported diabetes mellitus compared to 39.4% among those without self-reported diabetes mellitus (data not shown). Periodontal disease tends to worsen in the molar areas in patients with diabetes mellitus [22]. Moreover, a molar is often the first tooth lost in individuals with 28 teeth [23]. Therefore, patients with diabetes mellitus might have more tooth extractions due to periodontal disease when they have more teeth.

Regarding tooth type, patients with self-reported diabetes mellitus had significantly more upper anterior tooth extractions than those without self-reported diabetes mellitus (Table 3); however, such a difference was not observed for the other tooth types. Al-Shammari et al. [15] showed that anterior teeth were 3.23 times more likely to be extracted due to periodontal disease than posterior teeth. A possible explanation is that an early loss of molars might lead to mandibular anterior teeth pushing against the maxillary anterior teeth due to a decrease in the vertical dimension [24]. Consequently, anterior teeth and supporting bone are not able to maintain normal masticatory forces, and they begin to slant at an angle. It has been reported that occlusal trauma was associated with worsening periodontal disease [25]. Therefore, occlusal trauma might worsen the condition of periodontal disease in anterior teeth due to early loss of molars. Suzuki et al. [10] suggested that patients with diabetes mellitus lost their molar teeth at a younger age. However, we could not perform a detailed analysis because tooth types of the remaining teeth were not available for this survey.

Coelho et al. [6] indicated that several factors might be associated with dental caries in patients with diabetes mellitus, such as daily meal habits, salivary glucose, and low salivary flow. However, diabetes mellitus was not associated with tooth extraction due to dental caries in the current study. Although some studies [7,8] have reported an association between diabetes mellitus and dental caries, others [6] have reported no association. The relationship between diabetes mellitus and dental caries is not as strong as that of diabetes mellitus and periodontal disease. Therefore, tooth extraction due to dental caries may not be associated with diabetes mellitus.

This study has several limitations. Several confounding factors such as frequency of toothbrushing, dental visits, socio-economic status, and fluoride usage were not included in the analysis because the dentists participating in this study consisted of general practitioners who collected the data during routine dental procedures. Therefore, limited information was available for the analysis. The relatively low response rate may have caused selection bias affecting the study results. Caution should be exercised while generalizing our results to other populations because this study was conducted in Japan, which has universal health coverage. We used self-reported diabetes mellitus information. Although self-reported diabetes mellitus is reported as a valid measure of diabetes mellitus [16], the disease severity was not considered in this study. It is reasonable to assume that the risk of periodontitis among patients with well-controlled diabetes mellitus is not the same as those with poorly controlled diabetes mellitus. Therefore, clinical laboratory data is necessary to determine the disease severity. This study had a cross-sectional design; thus, a causal relationship was not established from the results.

## 5. Conclusions

Diabetes mellitus was significantly associated with tooth extraction due to periodontal disease even after adjusting for several confounders. Clinicians should keep in mind that periodontal disease might be a major risk factor for tooth extraction among patients with diabetes mellitus. In addition, tooth extraction in anterior teeth due to periodontal disease is more common in individuals with diabetes. This study strengthens the evidence for a relationship between diabetes mellitus and periodontal disease.

## Figures and Tables

**Table 1:** Characteristics of the extracted teeth in patients with and without self-reported diabetes mellitus.

Variable	Self-Reported Diabetes Mellitus				
	Yes (N = 455)		No (N = 4170)		*p*-Value ^a^
	N	%	n	%	
Sex					<0.001
Male	262	57.6	2001	48.0	
Female	193	42.4	2169	52.0	
Age (years)					<0.001
40–49	11	2.4	417	10.0	
50–59	42	9.2	728	17.5	
60–69	145	31.9	1188	28.5	
70–79	173	38.0	1148	27.5	
≥80	84	18.5	689	16.5	
Location					0.071
Rural	388	85.3	3411	81.8	
Urban	67	14.7	759	18.2	
Number of teeth before extraction					<0.001
<20	273	60.0	1994	47.8	
≥20	182	40.0	2176	52.2	
Smoking status					>0.999
Current smoker	92	20.2	842	20.2	
Nonsmoker	363	79.8	3328	79.8	
Tooth type					<0.001
Anterior	187	41.1	1349	32.4	
Premolar	134	29.5	1149	27.6	
Molar	134	29.5	1672	40.1	
Reason for tooth extraction					0.002
Dental caries	98	21.5	1026	24.6	
Periodontal disease	252	55.4	1947	46.7	
Others	105	23.1	1197	28.8	

^a^ *p*-values were calculated using chi-squared tests.

**Table 2:** Reasons for tooth extraction and number of teeth before extraction.

Reason for Tooth Extraction	<20 Teeth					≥20 Teeth				
	Self-Reported Diabetes Mellitus					Self-Reported Diabetes Mellitus				
	Yes (N = 273)		No (N = 1994)		*p*-Value ^a^	Yes (N = 182)		No (N = 2176)		*p*-Value ^a^
	n	%	n	%		n	%	n	%	
Dental caries	59	21.6	502	25.2	0.411	39	21.4	524	24.1	0.014
Periodontal disease	163	59.7	1119	56.1		89	48.9	828	38.1	
Other reasons	51	40.3	875	43.9		54	29.7	824	37.9	

^a^ *p*-values were calculated using chi-squared tests.

**Table 3:** Reasons for tooth extraction by self-reported diabetes mellitus status and tooth type.

Reason for Tooth Extraction	Anterior					Premolar					Molar				
	Self-Reported Diabetes Mellitus				*p*-Value	Self-Reported Diabetes Mellitus				*p*-Value	Self-Reported Diabetes Mellitus				*p*-Value
	Yes		No			Yes		No			Yes		No		
	n	%	n	%		n	%	n	%		n	%	n	%	
Upper					0.009					0.759					0.969
Dental caries	14	13.0	177	22.4		20	28.2	174	27.6		18	26.5	226	27.8	
Periodontal disease	70	64.8	393	49.7		30	42.3	244	38.7		33	48.5	384	47.3	
Others	24	22.2	221	27.9		21	29.6	213	33.8		17	25.0	202	24.9	
Total	108	100	791	100		71	100	631	100		68	100	812	100	
Lower					0.859										0.151
Dental caries	12	15.2	93	16.7		17	27.0	132	25.5	0.506	17	25.8	224	26.0	
Periodontal disease	58	73.4	393	70.4		29	46.0	209	40.3		32	48.5	324	37.7	
Others	9	11.4	72	12.9		17	27.0	177	34.2		17	25.8	312	36.3	
Total	79	100	558	100		63	100	518	100		66	100	860	100	

Chi-squared tests were performed to calculate the *p*-values.

**Table 4:** Association between self-reported diabetes mellitus and tooth extraction due to dental caries and periodontal disease.

	Model 1 ^a^			Model 2 ^b^			Model 3 ^c^			Model 4 ^d^		
	OR	95% CI	*p*-Value	OR	95% CI	*p*-Value	OR	95% CI	*p*-Value	OR	95% CI	*p*-Value
Dental Caries												
Diabetes mellitus												
No	1			1			1			1		
Yes	0.70	0.58–0.86	<0.001	0.87	0.71–1.06	0.169	0.87	0.71–1.06	0.171	0.85	0.68–1.06	0.14
Periodontal Disease												
Diabetes mellitus												
No	1			1			1			1		
Yes	1.417	1.17–1.72	<0.001	1.31	1.07–1.59	0.008	1.23	1.01–1.51	0.026	1.23	1.01–1.50	0.043

CI, confidence interval; OR, odds ratio. ^a^ Model 1: unadjusted. ^b^ Model 2: adjusted for sex, age, and location. ^c^ Model 3: adjusted for sex, age, location, number of teeth before extraction, and type of teeth. ^d^ Model 4: adjusted for sex, age, location, number of teeth before extraction, type of teeth, and smoking status.

## Data Availability

The data used from Japan’s second nationwide survey data collected by the 8020 Promotion Foundation is not publicly available. The datasets used and/or analyzed during the current study are available from the 8020 Promotion Foundation on reasonable request.
